# Salidroside can protect against ferroptosis in cardiomyocytes and may be related to the regulation of GGT1

**DOI:** 10.3389/fphar.2025.1580506

**Published:** 2025-05-14

**Authors:** Tianhang Feng, Jing Shi, Jinghua Zhao, Qin Zhao, Tao Wang, Sha Wan, Chen Fan, Sijia Wang, Chunyou Lai, Yutong Yao

**Affiliations:** ^1^ Department of International Medical, Sichuan Provincial Hospital, University of Electronic Science and Technology of China, Chengdu, China; ^2^ Science and Education Section, Hospital of Chengdu Office of People‘s Government of Xizang Autonomous Region (Hospital.C.X.), Chengdu, China; ^3^ Department of Biological Sample Bank, Hospital of Chengdu Office of People‘s Government of Xizang Autonomous Region (Hospital.C.X.), Chengdu, China; ^4^ Department of Cardiology, Hospital of Chengdu Office of People‘s Government of Xizang Autonomous Region (Hospital.C.X.), Chengdu, China; ^5^ Medical College, University of Electronic Science and Technology of China, Chengdu, Sichuan, China

**Keywords:** salidroside, ferroptosis, myocardial injury, Ggt1, cell death

## Abstract

**Indroduction:**

Ferroptosis, an iron-dependent cell death mechanism driven by lipid peroxidation, represents a novel therapeutic target for myocardial injury. Salidroside (SAL), a natural bioactive compound derived from Rhodiola rosea, exhibits cardioprotective effects through multi-target mechanisms with minimal adverse effects, yet its precise role in ferroptosis regulation remains unclear.

**Methods:**

This study systematically investigated SAL’s anti-ferroptotic effects using *in vitro* (RSL3-induced H9C2 cardiomyocytes) and *in vivo* (DOX-induced myocardial injury mouse model) approaches.

**Results:**

SAL treatment significantly enhanced cardiomyocyte viability by attenuating ferroptotic hallmarks, including lipid ROS accumulation, iron overload, lipid peroxidation, and mitochondrial dysfunction. Transcriptomic analysis revealed SAL-mediated modulation of DNA replication/repair, cell cycle regulation, protein autophosphorylation, drug ADME processes, and glutathione metabolism—a critical pathway in ferroptosis. Molecular docking identified γ-glutamyltransferase 1 (GGT1) as a high-affinity SAL target, linking drug metabolism and glutathione homeostasis. In MI mice, SAL downregulated GGT1 expression while restoring ferroptosis-related biomarkers: upregulating GPX4 and reducing SLC7A11/LC3II levels. Mechanistically, SAL suppresses ferroptosis through dual regulation of GGT1: (1) enhancing glutathione synthesis via GGT1 inhibition and (2) potentiating GPX4-mediated antioxidant defense.

**Discussion:**

These findings establish GGT1 as a pivotal therapeutic target for SAL’s cardioprotection, providing a mechanistic basis for its clinical application in ferroptosis-associated cardiovascular diseases.

## Introduction

Iron is an important trace element in the human body and participates in the catalytic reaction of intracellular enzymes. Iron deficiency reduces the physiological activity of iron-containing complexes, but excessive iron deposition causes excessive iron load in the body, which can catalyze the generation of high levels of reactive oxygen species (ROS), damaging cell membranes, intracellular proteins and nucleic acids and leading to various acute and chronic injuries (Silva et al., 2008). Studies have confirmed that excessive iron deposition in the body has a substantial toxic effect, and a large amount of ROS can initiate and regulate cell death under different pathological conditions (Le Borgne et al., 2017). Cell death plays an important role in growth and development, homeostatic regulation and pathological development of the body (Lin, 2003) and can be divided into regulated death and unregulated death (Guzmán, 2019). Among these processes, regulated death can be inhibited by specific pharmacological and genetic means, indicating that this process is regulated by specific molecular mechanisms (Cui et al., 2021). Ferroptosis is a newly defined type of regulated cell death (Guzmán, 2019).

Ferroptosis was identified by Brent R Stockwell. A nonapoptotic form of regulated cell death was first proposed by Stockwell et al., in 2012 (Dixon et al., 2012). Ferroptosis mainly depends on intracellular iron ions and is accompanied by the accumulation of intracellular lipid peroxides (Stockwell et al., 2017). In cells and mice, the selenoenzyme glutathione peroxidase (GPX4) is the key regulator of this form of cell death (Seibt et al., 2019). Ferroptosis is morphologically, biochemically, and genetically distinct from other types of cell death, such as necrosis, apoptosis, and autophagy. In terms of cell morphology, ferroptotic cells usually become round and are not connected to each other; mitochondria show some characteristic changes, mainly decreased mitochondrial volume, increased membrane density, reduction or disappearance of mitochondrial cristae, and mitochondrial outer membrane rupture (Dixon et al., 2012). In ferroptotic cells, nuclear changes are not obvious, and no chromatin marginalization or nuclear condensation is observed. In terms of biochemistry, ferroptosis can affect the metabolism of amino acids and glutathione (GSH), mainly by reducing the intake of intracellular cystine, depleting GSH, and inhibiting cystine/glutamate antiporter (System Xc) activity; thus, ferroptosis or inhibition of GPX4 enzymes can also directly lead to ferroptosis (Dixon et al., 2014). The ferroptosis activator RSL3 can directly inhibit GPX4 activity (Friedmann Angeli et al., 2014); thus, cells cannot use GSH to reduce lipid peroxides to nontoxic alcohols, resulting in strong accumulation of lipid peroxides and ferroptosis. Current research has shown that ferroptosis is closely related to many clinical diseases, including cancer (Timmerman et al., 2013), neurodegenerative diseases (Lane et al., 2021), brain diseases (Xiao et al., 2019), and ischemia‒reperfusion injury (Fang et al., 2019). Therefore, finding an effective way to regulate ferroptosis will help manage related clinical diseases.

Salidroside (SAL) has been identified as one of the most potent bioactive constituents isolated from various *Rhodiola* species, which mainly grow at altitudes of 1,600–4,000 m (Bai et al., 2019). Studies have shown that SAL has strong antioxidant and oxygen free radical scavenging effects and can increase the antioxidant capacity of cells (Yuan et al., 2013) and superoxide dismutase (SOD) and glutathione peroxidase (GSH-Px) activities to protect cells (Xu and Li, 2012). SAL has antiapoptotic effects on hypoxic cardiomyocytes and improves cardiac function by inhibiting the enzymatic activity of caspase-3 (Wu et al., 2019).

H9c2 cells, derived from rat embryonic ventricular myocardium, were a well-established *in vitro* model for studying cardiomyocyte pathophysiology. Their advantages include: Retention of cardiac-specific markers (e.g., LC3Π) and functional ion channels, susceptibility to lipid peroxidation and iron overload, aligning with our focus on ferroptosis regulation (Chen et al., 2017). SAL also protected H9C2 cells from H_2_O_2_-induced oxidative damage by reducing intracellular ROS and malondialdehyde (MDA) levels and increasing SOD activity (Gao et al., 2021). However, no reports have examined the effects of SAL on ferroptosis. Therefore, in this study, we carried out an exploratory study on the effect of SAL on ferroptosis.

Herein, we designed the present study to further investigate the effect and mechanisms by which SAL regulates ferroptosis, aiming to shed new light on ferroptosis prevention by natural phytochemicals.

## Materials and methods

### Chemicals and reagents

SAL was purchased from Chengdu Refines Biotechnology Co., Ltd. (Chengdu, China). Anti-GPX4 (ab125066), anti-SLC7A11 (ab175186) and anti-cTNT (ab209813) were obtained from Abcam, and anti-GGT1 (YT5266) rabbit monoclonal antibody and anti-GAPDH (AF7021) rat monoclonal antibody were obtained from Affinity. Anti-LC3Π (ET1701-65) was purchased from Huabio. The CCK-8, GPX4 and GGT1 assay kits were all purchased from Shanghai Lianmai Bioengineering Co., Ltd. An Fe assay kit was purchased from Elabscience, and a BCA quantification kit was purchased from Biosharp. The MDA assay kit, Cys assay kit, and Glu assay kit were purchased from Nanjing Jiancheng Bioengineering Institute. Corresponding experiments were performed according to the manufacturer’s instructions to measure the levels of each substance in each group of H9c2 cardiomyoblasts.

### Cell culture

The H9c2 cardiomyoblast cell line (Henan Province Industrial Microorganisms Engineering Technology Research Center) was propagated in DMEM growth medium containing 10% fetal bovine serum with dual antibiotics: 100 μg/mL streptomycin and 100 U/mL penicillin. Cellular maintenance occurred in a controlled 37°C environment with 5% CO2 saturation. Subculturing was performed using trypsin/EDTA digestion when 80%–90% confluency was attained, followed by centrifugation and resuspension in fresh complete medium for subsequent passages.

### Cell treatment

Cultured H9c2 cells that reached 80%–90% confluence from passages 3–10 were used for experiments. Cultured H9c2 cells were divided into the following three groups: (i) the control group (NC, n = 5), (ii) the RSL3 group (RSL3, n = 5), and (iii) the RSL3 + SAL group (SAL, n = 5). The RSL3 group was the ferroptosis group, and the cells were incubated with 2 µM RSL3 for 24 h, the concentration and time of induce were adopted based on established methodologies from Wang et al. (2024). In addition, we performed CCK-8 assays to evaluate cellular viability across a gradient of SAL concentrations (0.4–4 mg/mL). Then the SAL group was exposed to 4 mg/mL SAL for 4 h before 2 µM RSL3 induction for 24 h. The treatment duration rationale: the 24-h intervention protocol was adopted based on established methodologies from Ju et al. (2024), which identified this timeframe as critical for maximal ferroptosis suppression in cardiomyocytes. RSL3 and SAL were diluted with medium. At the end of the incubation, the cells were harvested and processed for analysis by RNA sequencing and subsequent detection.

### Lipid ROS production assay

For redox status evaluation, H9c2 cells (2 × 105 cells/well in 6-well plates) were subjected to BODIPY™ C11 (5 μM) labeling post-treatment. Following overnight DMEM incubation, cells underwent 30-min probe loading at 37°C. Post-labeling procedures included: (1) triple PBS washing to remove excess dye, (2) enzymatic detachment using 0.25% trypsin, and (3) fluorescence analysis *via* FACSCalibur™ flow cytometer (BD Biosciences) with simultaneous detection at 488/530 nm (green) and 488/585 nm (red) channels.

### MDA, Glu, Fe^2+^ and Cys assays

The MDA, Glu, Fe^2+^ and Cys contents in each group of H9c2 cardiomyoblast cells were assessed using the corresponding assay kit and are presented as µmol per milligram of protein according to the manufacturer’s protocol.

### Transcriptome sequencing

Transcriptomic profiling was performed with five biological replicates per experimental group (n = 5) to minimize inter-group variability. Total RNA was extracted from H9c2 cardiomyocytes using TRIzol reagent. RNA sequencing procedures commenced with cellular harvesting through cold PBS rinsing (3 cycles, 4°C). Polyadenylated mRNA enrichment was achieved *via* poly-T magnetic bead selection (Illumina TruSeq PE Cluster Kit protocol) following total RNA extraction. Integrity verification (RIN >8.0) preceded cDNA library construction through PCR amplification (KAPA HiFi HotStart ReadyMix). Raw sequencing reads underwent quality control filtration using Trimmomatic v0.39 to eliminate adapter sequences and low-quality bases (Phred score <30). Indexed samples were clustered on cBot systems (TruSeq PE Cluster Kit v3-cBot-HS) under standardized thermal cycling conditions (Illumina technical manual). Paired-end sequencing (150 bp reads) was performed on NovaSeq 6,000 platforms, generating approximately 40 million reads per sample. Post-sequencing processing included: ① HISAT2 v2.0.5 alignment to reference genome GRCh38 ② Transcript quantification *via* FeatureCounts v1.5.0-p3 ③ FPKM normalization accounting for gene length and sequencing depth ④ Differential expression analysis using Cufflinks’ expectation-maximization algorithm (default parameters). Functional annotation employed Metascape platform (v2023.05) with KEGG pathway enrichment (FDR <0.05).

### Quantitative real-time PCR

To validate transcriptional analysis of DEGs, we selected gene GGT, a key gene involved in glutathione metabolism and the drug ADME for qRT-PCR confirmation. Primer sequences that Forward 5′-CTG​GGG​AGA​TCC​GAG​GCT​AT-3′, Reverse 5′-GAT​GAC​GGT​CCG​CTT​GTT​TTC-3' (amplicon size: 152 bp) were designed using Primer-BLAST (NCBI) and validated for amplification efficiency (90%–110%). β-actin and GAPDH served as reference genes, with expression levels normalized using the 2^(-ΔΔCt) method. Statistical consistency between RNA-seq and qRT-PCR results was assessed *via* Pearson correlation analysis (r = 0.87, p < 0.001).

### Molecular docking

Molecular docking simulates ligand-receptor interactions by computationally predicting binding affinity and optimal conformations through physicochemical parameter optimization. The protocol comprised three phases: First: receptor preparation (GGT1 crystal structure (PDB ID: 6XPB) was retrieved from the RCSB Protein Data Bank); second:ligand preparation (Salidroside 3D structure (PubChem CID: 159278) was downloaded in SDF format); finally, protein optimization was carried out that① Water molecule removal ② Polar hydrogen addition ③ Partial charge calculation using Gasteiger method. Ligand-receptor docking was executed in AutoDock Vina 1.2.2 with search space dimensions 30 × 30 × 30 Å (grid spacing 0.05 nm). Conformational sampling parameters: exhaustiveness = 20, num_modes = 9. PyMOL v2.5.4 generated visualization outputs, with binding poses ranked by affinity scores (kcal/mol). The most favorable conformation was selected based on cluster analysis (RMSD cutoff = 2.0 Å).

### Animal experiment

Animal protocols (Ethics Approval: KY 2020-39) utilized 8-week-old male C57BL/KS mice (Jicui Yaokang Biotech) in temperature-controlled SPF conditions (22°C ± 1°C, 12 h light cycle). Experimental groups (n = 10/group): ① WT control ② DOX-induced myocardial injury (20 mg/kg i. v.) ③ SAL pretreatment (1.5 g/kg i. p.) + DOX. Drug administration timelines: SAL pretreatment (−24 h) → DOX challenge (0 h) → terminal procedures (72 h post-DOX). The vivo dose of 1.5 g/kg SAL was depend on our preliminary studies (Shi et al., 2022), where it significantly attenuated ferroptosis markers (GPX4↑) in a rat diabetes model. Terminal blood collection via cardiac puncture preceded rapid freezing (−80°C) of serum and cardiac tissue. Euthanasia followed AAALAC guidelines: CO2 asphyxiation (30% chamber volume/min) → cervical dislocation confirmation.

#### Determination of biochemical parameters

Serum levels of iron were determined by the Hospital of the Chengdu Office of the People’s Government of the Tibetan Autonomous Region using the corresponding colorimetric kit according to the manufacturer’s instructions.

#### Pathological analysis

Formalin-fixed paraffin sections (3 μm) underwent H&E and Prussian blue staining. Histopathological scoring (200×, Nikon Eclipse) followed three-tier grading: 0 = normal architecture; I = focal subendocardial necrosis; II = multifocal transmural lesions; III = confluent pan-cardiac damage. Panoramic scanning (3DHISTECH Pannoramic MIDI) enabled whole-slide digital analysis.

#### MDA and GSH assays

The MDA and GSH contents in each group of mice were assessed using the corresponding assay kit and are presented as µmol per milligram of protein according to the manufacturer’s protocol.

#### Western blot analysis

Cardiac lysates (RIPA buffer, 1:10 w/v) underwent centrifugation (12,000g, 10 min, 4°C). BCA-quantified proteins (20μg/lane) were resolved on 10% SDS-PAGE and transferred to PVDF membranes (0.45 μm). Blocking (5% non-fat milk/TBST, 1 h) preceded overnight incubation with primary antibodies (4°C) and HRP-conjugated secondary probing (1 h, RT). Chemiluminescent detection (ECL Plus) employed ChemiScope 6100 with densitometric analysis normalized to GAPDH (Image Pro-Plus 6.0).

### Transmission electron microscopy

H9c2 cardiomyoblast cells and heart tissue from each group were removed and fixed in glutaraldehyde (3%), after which they were fixed in 1% osmium tetroxide. Subsequently, the samples were dehydrated step by step, permeated, and embedded in Araldite. Then, the tissues were cut into ultrathin sections (50 nm) using an ultramicrotome (Leica EM UC7, Solms, Germany) and transferred to a copper net. Subsequently, the thin slices on the copper net were stained with uranyl acetate (2%) and lead citrate at room temperature for 15–20 min and observed under a transmission electron microscope (JEM-1400FLASH, JEOL, Japan).

### Statistical analysis

All measurements expressed as mean ± SD (n ≥ 3). Intergroup differences assessed *via* one-way ANOVA with Tukey’s correction (SPSS 21). Graphical representations generated in Prism 8 (GraphPad), with P < 0.05 considered significant.

## Results

### Protective effect of SAL on RSL3-induced cardiomyocyte ferroptosis

**FIGURE 1:**
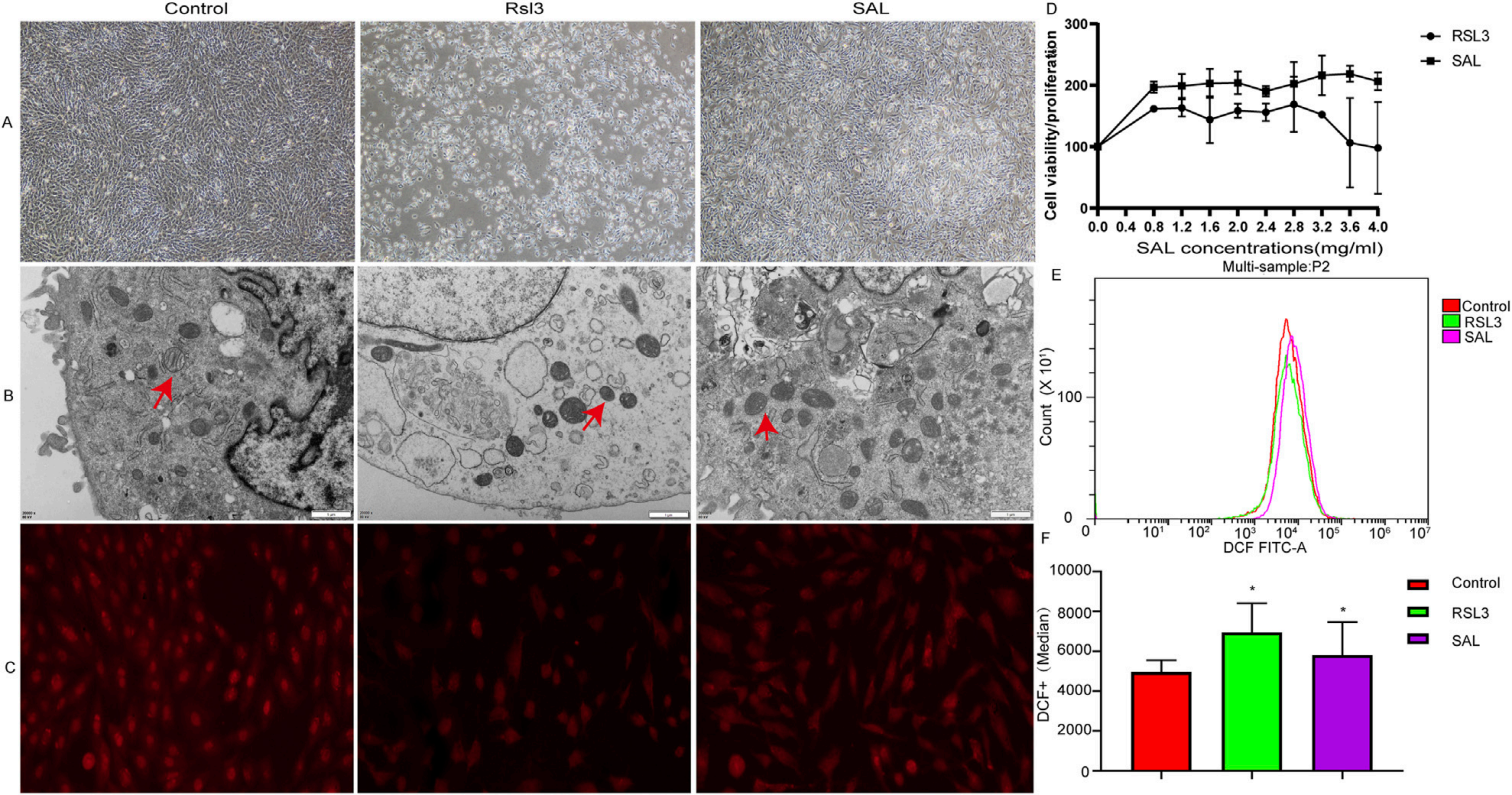
Protective effect of SAL on RSL3-induced ferroptotic cardiomyocytes. **(A)** Electron microscope to detect cardiomyocytes in each group Morphology; **(B)** TEM to detect mitochondrial morphological changes under electron microscope in each group; **(C)** Immunofluorescence detection of GPX4 protein expression in cardiomyocytes of each group; **(D)** CCK8 to detect the cell proliferation level of cardiomyocytes in each group; **(E,F)** Flow cytometry to detect the level of ROS in cardiomyocytes in each group (*p < 0.05 as SAL and RSL3 group vs. Control group).

To explore the protective effect of SAL on ferroptotic cardiomyocytes, the cells in the SAL-treated group were pretreated with SAL for 4 h, and after 24 h, ferroptosis was induced by RSL3 for 24 h. Then, we assessed various physiological indicators of cardiomyocyte ferroptosis. The biochemical characteristics of ferroptosis mainly include the accumulation of ROS and the inhibition of the cystine/glutamate anti-transport system (System Xc-) pathway (Zhao et al., 2021a). Here, we report that ferroptosis plays an important role in RSL3-induced cardiomyocyte damage. General morphological observation revealed that the cardiomyocytes in the RSL3 group were significantly atrophied, and the cell density was reduced. The cardiomyocytes in the SAL group were more similar to those in the normal group, and the cell density was greater than that in the RSL3 group (Figure 1A). The morphological changes in the mitochondria of the cardiomyocytes in each group were observed by transmission electron microscopy. Some mitochondria in the cytoplasm in the RSL3 group exhibited pyknosis, while the morphological structure of the mitochondria in the SAL group was complete and clear (Figure 1B). Immunofluorescence analysis of the ferroptosis marker GPX4 in cardiomyocytes revealed that GPX4 levels decreased after RSL3-induced ferroptosis and increased in the cardiomyocytes pretreated with SAL (Figure 1C). Many studies have shown that ferroptosis is initiated when GPX4 activity is inhibited (Friedmann Angeli et al., 2014; Sakai et al., 2017). Both GPX4 immunofluorescence and mitochondrial electron microscopy indicated that SAL significantly alleviated the RSL3-induced ferroptosis. CCK-8 cell viability assays revealed that the cardiomyocytes in the RSL3 group were more sensitive to RSL3-induced cell death than were the cardiomyocytes in the SAL group (Figure 1D). Concurrently,the ROS level in the SAL group was lower than that in the RSL3 group, and all higher than control group (Figures 1E,F), which was consistent with the results of other studies (Li et al., 2020). Changes in mitochondrial morphology are a distinctive feature of ferroptosis (Koenders et al., 2019). Taken together, these results indicate that SAL effectively prevents RSL3-induced ferroptosis. These observations suggest that SAL plays an important role in preventing RSL3-induced cardiomyocyte ferroptosis.

### SAL attenuates RSL3-induced ferroptosis by inhibiting iron accumulation and lipid peroxidation

**FIGURE 2:**
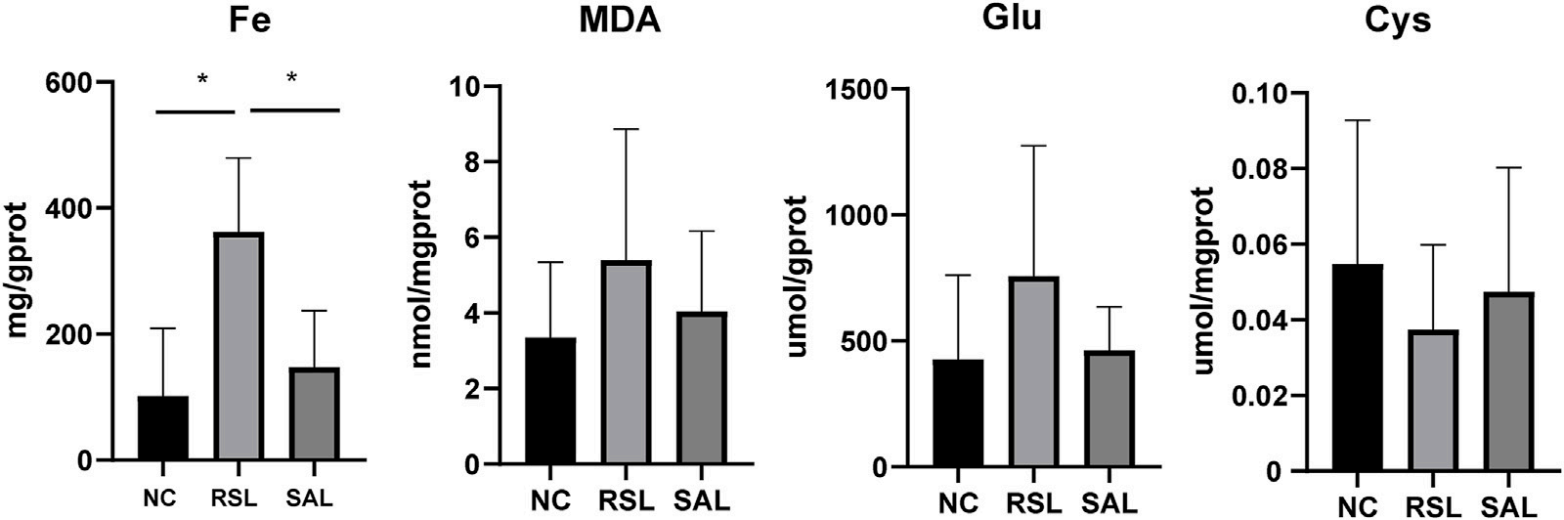
The levels of iron accumulation and lipid peroxidation in each group.

To further confirm the effect of SAL on ferroptosis, we determined the levels of Fe, Cys, Glu, and MDA in each group of cells. The level of Fe in the RSL group was significantly greater than that in both the NC group (P < 0.001) and the SAL group (P < 0.001). The levels of Glu and MDA in the RSL3 group were greater than those in the SAL and NC groups. However, the level of Cys in the RSL3 group was lower than that in the SAL group (Figure 2). These results suggested that SAL may alleviate the extent of cardiomyocyte ferroptosis by regulating iron levels and lipid peroxidation.

### SAL alters transcription in RSL3-induced ferroptotic cardiomyocytes

**FIGURE 3:**
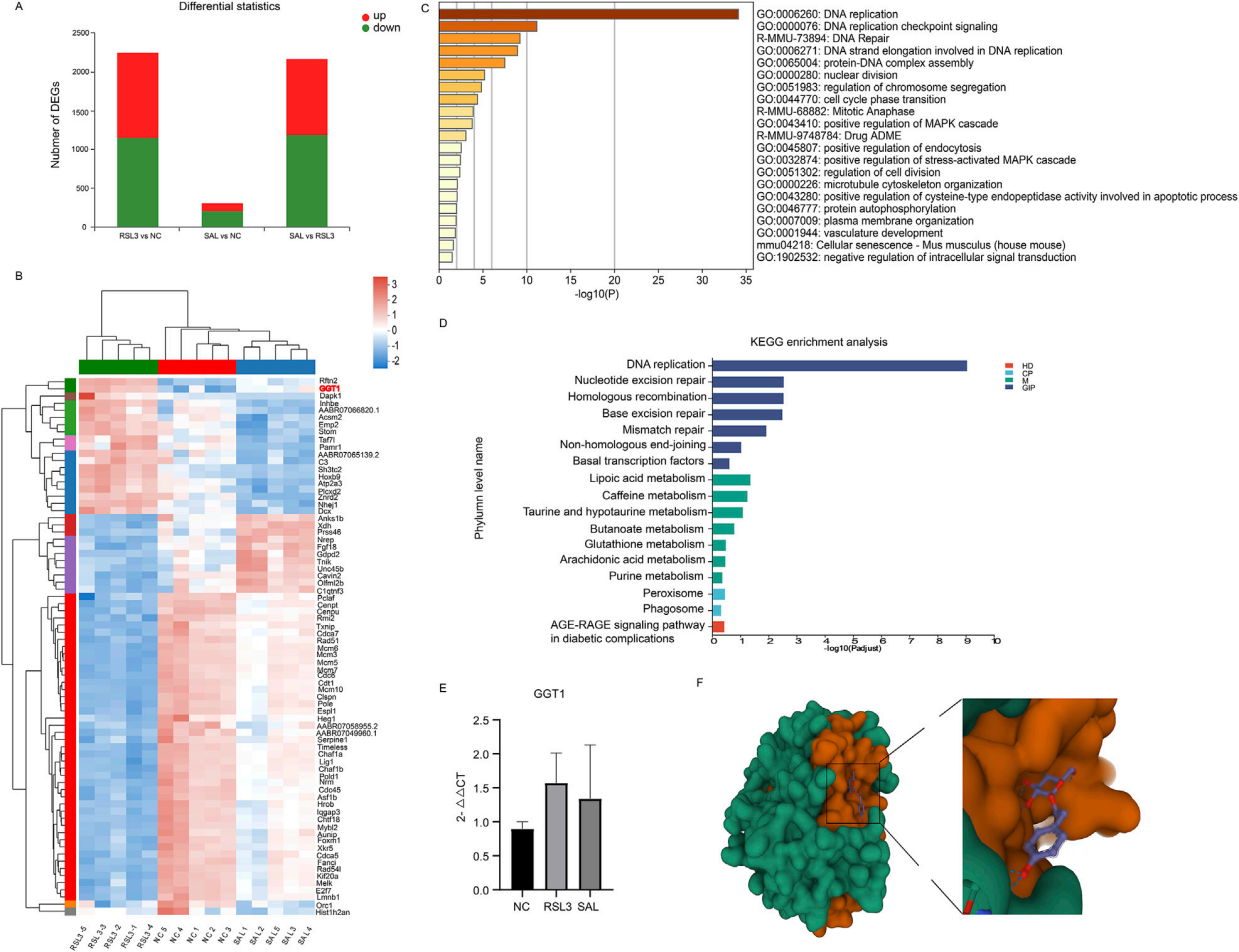
**(A)** Transcriptional distribution of DEG in cardiomyocytes of each group; **(B)** Heatmap representation of hierarchical clustering of gene expression levels for 74 core DEGs; **(C)** Functional enrichment results of 74 core DEG regulated by both RSL3 and SAL on the METASCAPE website; **(D)** Pathways associated with 74 core DEGs were enriched by KEGG analysis. Different colors represent the branches of the KEGG metabolic pathway, M stands for metabolism, GIP stands for genetic information processing, CP stands for cellular process, and HD stands for human disease; **(E)** QPCR of the expression level of GGT1 in cardiomyocytes of each group; **(F)** PyMOL software The three-dimensional spatial pattern of the output SAL and target GGT1 molecular docking.

We used transcriptome sequencing to study the mRNAs specifically regulated by RSL3 and SAL and estimated their impact on cardiomyocyte function through the transcriptome. Differentially expressed genes (DEGs) between groups were screened with |log 2 FC| > 1 as the cutoff criterion and q-value <0.05 as the criterion. There were 2,252 DEGs in the RSL3 group and NC group; 1,101 DEGs were upregulated, and 1,151 DEGs were downregulated (Figure 3A). The SAL group and RSL3 group had a total of 2,174 DEGs, of which 979 DEGs were upregulated and 1,159 DEGs were downregulated. There were 309 DEGs in the SAL group and the NC group; 104 DEGs were upregulated, and 205 DEGs were downregulated. A Venn diagram was used to visualize the intersection of DEGs, and a total of 77 common DEGs were identified among the three groups. Among these DEGs, 55 decreased after ferroptosis induction and increased after SAL treatment; 19 increased after ferroptosis induction and decreased after SAL treatment (Appendix Table S1). This finding suggested a potential protective role of these DEGs in the prevention and treatment of ferroptosis. We believe that the 74 DEGs identified are the main targets of SAL for the treatment of cardiomyocyte ferroptosis.

### GGT1 may be a potential ferroptosis target of SAL

Two enrichment analyses were performed on the 74 DEGs of interest *via* Metascape and KEGG. The 74 DEGs regulated by both RSL3 and SAL were subjected to functional enrichment analysis on the Metascape website. The DEGs regulated by RSL3 and SAL were mainly related to DNA replication, DNA repair, the cell cycle, protein autophosphorylation, and the drug absorption, distribution, metabolism, and excretion (ADME) (Figure 3C). These findings suggest that these pathways are affected by RSL3 treatment of cardiomyocytes and by the effects of SAL on ferroptotic cardiomyocytes, which ameliorates their cellular damage. The ADME of traditional Chinese medicine after administration all have a major impact on its biological effects. SAL has specific pharmacokinetic properties in rats with myocardial ischemia (Gu et al., 2018), so the drug ADME (R-MMU-9748784) pathway plays an important role in ferroptotic cardiomyocytes treated with SAL. In this study, the DEGs involved in the drug ADME pathway (R-MMU-9748784) were GGT1, ACSM2 and XDH. Pathways associated with 74 important DEGs were enriched according to KEGG analysis. KEGG analysis revealed that the GSH metabolism pathway was affected by RSL3 and SAL (Figure 3D). GSH metabolism is involved in the regulation of ferroptosis initiation and execution (Wu et al., 2021).

By consulting the previous research of GGT1, ACSM2 and XDH, we found that GGT plays a key role in GSH metabolism (Nakajima et al., 2013). One important role is to cleave extracellular GSH, which is used as a Cys source for GSH biosynthesis; another important physiological role is to cleave GSH peptide-S-conjugates as a key step in xenobiotic detoxification and drug metabolism (Nakajima et al., 2013). In our study, the GGT1 gene, encoding GGT, was involved in GSH metabolism. The expression level of GGT1 was significantly different among the groups (Figure 3B) and showed a trend of first increasing and then decreasing; the same trend was also found in the qPCR results of GGT1 in cardiomyocytes (Figure 3E). Therefore, we considered GGT1 to be the most important gene involved in ferroptosis and SAL treatment in this study, which has implications for the treatment of ferroptosis-associated diseases.

### Interaction between SAL and the GGT1 receptor

To further demonstrate the potential impact of SAL on key putative targets, we performed molecular docking analysis of SAL (CID: 159,278) and GGT1 (PDB: 6XPB) *via* Autodock Vina 1.2.2 software. The active substance *Rhodiola* astragaloside binds to its protein (6XPB) target *via* visible hydrogen bonds and strong electrostatic interactions. According to the molecular docking results (Figure 3D), the conformational energy required for the binding of SAL to the target protein is low (−5.831 kcal/mol), indicating that SAL has a good binding ability to GGT1.

### SAL alleviates pathological changes in myocardial injury models

**FIGURE 4:**
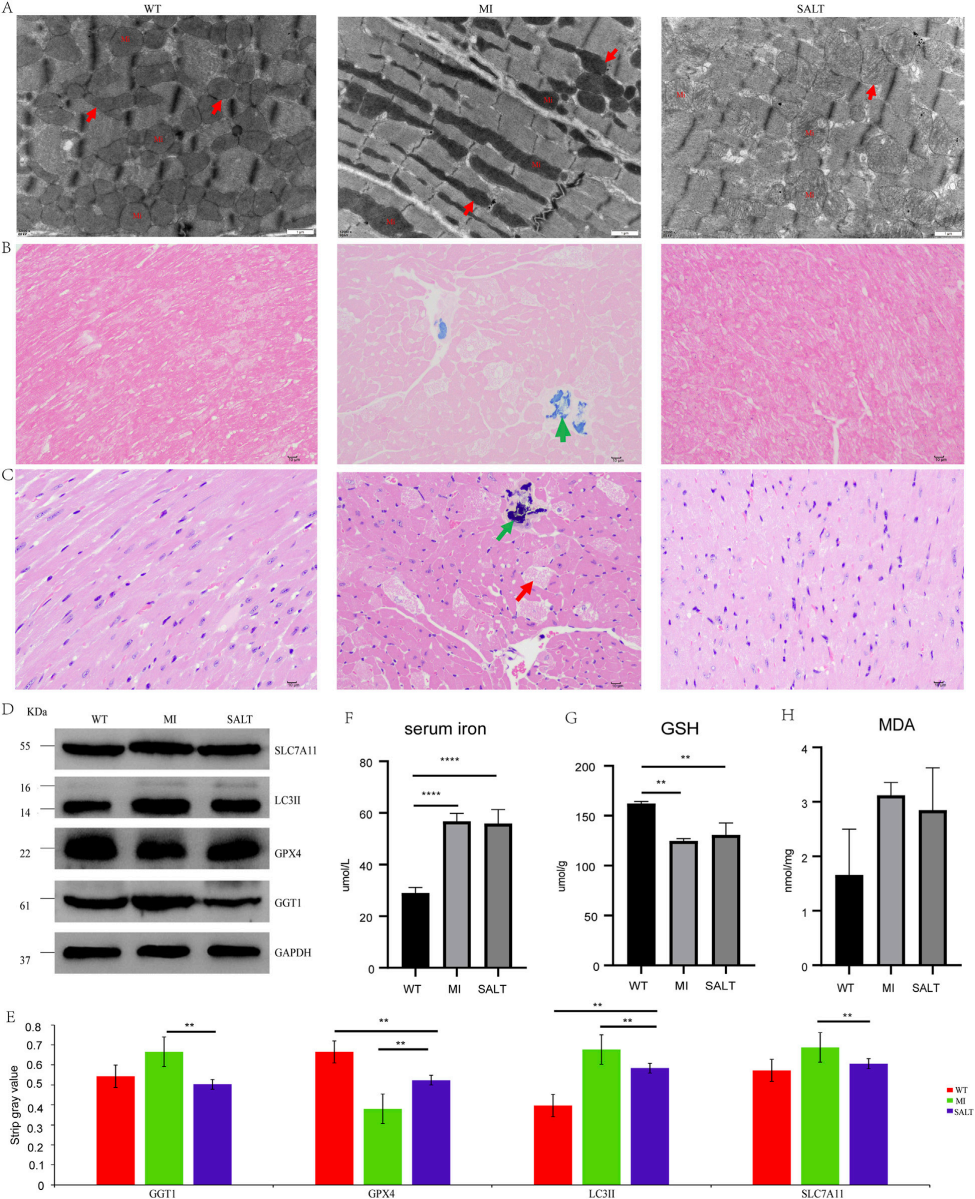
**(A)** Cardiomyocyte mitochondria under electron microscope in each group of mice (×12,000). Mi: mitochondria.↑indicates that normal mitochondria or mitochondrial pyknosis or mitochondrial swelling. **(B)** Prussian blue staining of myocardial tissues of mice in each group (×400).↑indicates positive expression of iron or hemosiderin. **(C)** HE staining of myocardial tissue in each group of mice (×400). ↑ indicates that myocardial fibrosis and necrosis.↑indicates that aggregation of basophils. **(D)** WB result in different groups of SLC7A11, LC3II, GPX4, and GGT1. **(E)** The strip Gy value of SLC7A11, LC3II, GPX4, and GGT1 of each group. **(F)** The level of serum iron of each group.G.The level of GSH of each group. **(H)** The level of MDA of each group. Ns:**P < 0.005, compared with WT group,****P < 0.0001,compared with WT group.

To assess the effects of SAL under conditions of cardiac oxidative stress *in vivo*, we utilized a mouse model of acute myocardial injury. Transmission electron microscopy was used to observe the mitochondrial morphology and ferroptosis of mouse cardiomyocytes in each group. According to the changes in mitochondrial morphology, the trend of lesions may be as follows: WT group < SALT group < MI group (Figure 4A). Compared with those in the NC group, the cardiomyocytes in the MI group exhibited obvious abnormalities, regular arrangement of myofibrils, an intact dark band, and mitochondrial pyknosis; moreover, a few lipid droplets were observed in the cytoplasm. The cardiomyocytes of the mice that were previously administered SAL exhibited mild abnormalities. These findings suggest that SAL can effectively attenuate myocardial injury-induced mitochondrial damage. To further evaluate the effect of SAL on the iron content in the heart, we performed Prussian blue staining on the heart tissues of the mice in each group. The Prussian blue staining data showed that DOX strongly induced the accumulation of iron ions in mouse heart tissue, and SAL pretreatment attenuated the iron deposition (Figure 4B).

To further observe the therapeutic effect of SAL on myocardial injury in mice, we performed H&E staining on the heart tissues of the mice in each group. H&E staining revealed that the degree of cardiac lesions in the MI group was relatively severe, as indicated by myocardial fibrosis and necrosis and the accumulation of basophilic substances (Figure 4C).

### SAL alters protein expression in myocardial injury models

To monitor myocardial damage, we determined SLC7A11 and LC3II protein expression levels (Figure 4D). The protein expression levels of myocardial injury markers in the mice with MI were significantly increased, and the levels were significantly decreased after administration, suggesting that SAL could alleviate myocardial injury in the mice with MI. Western blotting revealed variable expression of the ferroptosis marker GPX4 and the key protein GGT1 in the heart tissue of each group (Figure 4D). The strip gray value of SLC7A11, LC3II, GPX4, and GGT1 of each group in Figure 4E. At the protein level, the ferroptosis-related factor GPX4 showed a relative decrease in expression, while GGT1 expression increased in the MI group. The expression of GPX4 and GGT1 in the heart tissue of the SAL-pretreated mice approached that in the heart tissue of the control mice. The *in vitro* results are consistent with the *in vivo* results. SAL significantly reduced ferroptosis and oxygen damage in the cardiac tissue of the mice with myocardial injury. Collectively, our findings confirm the involvement of ferroptosis in myocardial injury and reveal that SAL may prevent myocardial injury by inhibiting ferroptosis through the regulation of GGT1.

### SAL alleviates iron deposition and lipid peroxidation in myocardial injury models

To further confirm the effect of SAL on ferroptosis in mice, we measured the levels of serum iron, MDA and GSH in each group in our study. The serum iron concentration in the MI group was slightly greater than that in the SALT group but was significantly greater than that in the WT group (P < 0.0001) (Figure 4F). The level of GSH was lower in the MI group than in the SALT group and WT group (Figure 4G). However, the MDA concentration in the MI group was greater than that in the SALT group and WT group (Figure 4H). These findings suggested that SAL might alleviate the extent of myocardial injury-related ferroptosis by regulating lipid peroxidation.

## Discussion

Myocardial injury represents a central pathological event in the progression of cardiovascular diseases, including myocardial infarction, ischemia-reperfusion injury, chemotherapeutic agent-induced cardiotoxicity (e.g., doxorubicin), and pressure overload conditions (Songbo et al., 2019). The pathogenesis involves a multifaceted dysregulation of signaling pathways, primarily characterized by inflammatory cytokine release, energy metabolism disturbances, and oxidative stress. Among these, oxidative stress emerges as the predominant driver, wherein excessive reactive oxygen species (ROS) disrupt mitochondrial electron transport chain function, resulting in lipid peroxidation, protein misfolding, and DNA damage. Targeted inhibition of oxidative stress has therefore emerged as a promising therapeutic strategy to mitigate myocardial damage (Li et al., 2023).

Notably, Ferroptosis, a novel form of regulated cell death, is characterized by iron-dependent lipid peroxidation, collapse of antioxidant defense systems, and dysregulated iron metabolism. These pathophysiological hallmarks are closely associated with the molecular mechanisms underlying myocardial injury (Tang et al., 2021). Recent studies demonstrate that ferroptosis amplifies cardiomyocyte death through three interconnected axes: (1) iron overload *via* TFR1-mediated iron uptake and NCOA4-dependent ferritinophagy, (2) GPX4 inactivation leading to glutathione depletion and impaired antioxidant defense, and (3) ACSL4/LOX-mediated lipid peroxidation that destabilizes cellular membranes. Targeted inhibition of ferroptosis thus represents a promising cardioprotective strategy for both the treatment and prevention of cardiovascular diseases, offering a multimodal approach to restore redox homeostasis and mitigate iron-mediated cellular damage (Cheng et al., 2024).

Salidroside, was primarily isolated from the traditional medicinal herb Rhodiola rosea. Modern pharmacological investigations have elucidated salidroside’s multifaceted therapeutic properties, including antioxidant and anti-inflammatory effects, mitochondrial protection, metabolic regulation, and anti-apoptotic/anti-necrotic activities (Liang et al., 2024). Some studies have demonstrated that salidroside exerts cardioprotective and neuroprotective effects by modulating iron metabolism homeostasis (Beretta, 2024). Furthermore, it activates the GPX4 antioxidant axis *via* Nrf2 nuclear translocation, thereby restoring the glutathione (GSH) antioxidant system to counteract ferroptotic damage (Yang et al., 2023). These mechanisms collectively position salidroside as a potent regulator of redox balance and iron-dependent cell death pathways in pathological contexts.

In our study, gene transcriptional changes were detected between groups of cardiomyocytes and were attenuated after early SAL intervention in ferroptotic cells. SAL has a wide range of pharmacological properties, and current pharmacokinetic studies on SAL have focused mainly on its absorption properties (Zhang et al., 2021). The functional enrichment of DEGs regulated by both RSL3 and SAL showed that the protective effect of SAL on ferroptotic cells can be exerted by regulating DNA replication, the cell cycle, protein autophosphorylation, drug ADME and GSH metabolism. At the cellular level, ferroptosis is primarily driven by iron-dependent lipid peroxidation. In addition to iron metabolism, Cys deficiency and GPX4 inactivation have been shown to promote ferroptosis (Stockwell et al., 2017). In this study, cardiomyocytes induced by RSL3 presented a typical ferroptotic state, the iron metabolism-related indicator Fe increased in the RSL3 group, ROS were also the highest in the RSL3 group, mitochondrial pyknosis occurred in the RSL3 group, and lipids and the contents of peroxidation indicators (GPX4, Cys, MDA, Glu) changed, although there were no significant difference in the statistical analysis of MDA, Glu and Cys in our study, this may be related to the uneven absorption of our supernatant or the accuracy of the detection kit, but the overall results, cell morphology and electron microscope results were all consistent with the results of many studies (Zhao et al., 2021b; Sui et al., 2018; Fang et al., 2022; Wang et al., 2020). In the cardiomyocytes of the myocardial injury model mice in this study, mitochondrial pyknosis, ferroptosis, increased levels of the ferroptosis markers SLC7A11 and LC3II, and decreased expression of the ferroptosis regulatory gene GPX4 protein were observed, indicating a pattern typical of ferroptosis. In our previous study, SAL was shown to have a regulatory effect on iron metabolism in diabetic mice (Shi et al., 2022), and ferroptosis of myocardial cells with myocardial injury was significantly alleviated after SAL intervention, suggesting that SAL may regulate iron metabolism by regulating iron metabolism to protect ferroptotic cardiomyocytes. The same conclusion was also obtained in the cell model and mouse model in this study. SAL reduced ferroptosis-induced myocardial damage and lipid peroxidation and increased GGT1 expression.

GGT1 was one of the most significantly DEGs among the 74 core genes. GGT1 is the core gene in the drug ADME pathway, and the GGT dimer hydrolyzes GSH conjugates and then forms cysteinylglycyl conjugates (Cys-Gly) and glutamate (LGlu) (Paracetamol ADME, 2025), suggesting that GGT1 may also play the same role in SAL ADME, which requires subsequent experimental verification. According to our molecular docking results, GGT1 and SAL bind stably, indicating that GGT1 may be a potential drug target of SAL to protect injured cardiomyocytes. Numerous studies have shown that high levels of GGT1 are associated with cardiovascular risk (Turgut et al., 2006), heart failure (Wang et al., 2013), and myocardial infarction (Ozcan et al., 2012), which is consistent with the findings of this study. Studies have shown that drug inhibition or deletion of GGT1 inhibits cell density-induced increases in intracellular GSH levels and cell viability under cystine deprivation, while the addition of cysteine (the GGT product of GSH cleavage) can restore cell density (Hayashima and Katoh, 2022). The GSH level and cysteine content are closely related to the occurrence of ferroptosis, indicating that the expression of GGTI may be related to ferroptosis. In cardiomyocytes pretreated with SAL, the level of GGT1 was significantly lower, suggesting that SAL may have a protective effect on cardiomyocytes undergoing ferroptosis by binding to the receptor of GGT1.

In this study, we investigated the cardioprotective effects of salidroside (Sal) against RSL3-induced myocardial injury. Our results demonstrate that Sal significantly attenuates intracellular ROS accumulation and mitochondrial ferroptosis, concurrently reducing cellular levels of Fe^2+^, cysteine (Cys), glutamate (Glu), and malondialdehyde (MDA). Immunofluorescence and Western blotting analyses revealed that Sal robustly upregulates GPX4 expression in injured cardiomyocytes, reinforcing glutathione-dependent antioxidant defenses. Transcriptomic profiling of Sal-treated H9C2 cells further uncovered its multi-target regulatory actions, including modulation of DNA replication/repair, cell cycle progression, protein autophosphorylation, drug ADME processes (absorption, distribution, metabolism, excretion), and glutathione metabolism. Network pharmacology and molecular docking analyses identified gamma-glutamyltransferase 1 (GGT1) as a potential therapeutic target, wherein Sal-mediated GGT1 upregulation correlates with its cardioprotective efficacy.

These findings collectively highlight Sal’s ability to orchestrate a tripartite regulatory network—spanning iron homeostasis, lipid peroxidation suppression, and antioxidant reactivation—to exert multi-layered inhibition of ferroptosis. The identification of GGT1 as a key mediator positions Sal as a promising candidate for clinical translation in cardiovascular diseases. Future studies should focus on elucidating Sal’s subcellular targeting specificity and long-term safety. This work provides both mechanistic insights and a robust framework for developing ferroptosis-targeted therapies against myocardial injury diseases.

## Data Availability

The raw sequence data reported in this paper have been deposited in the Genome Sequence Archive (Genomics, Proteomics & Bioinformatics 2021) in National Genomics Data Center (Nucleic Acids Res 2022), China National Center for Bioinformation/Beijing Institute of Genomics, Chinese Academy of Sciences (GSA: CRA022740) that are publicly accessible at https://ngdc.cncb.ac.cn/gsa.
